# A study on the evaluations of emission factors and uncertainty ranges for methane and nitrous oxide from combined-cycle power plant in Korea

**DOI:** 10.1007/s11356-012-1144-1

**Published:** 2012-09-22

**Authors:** Seehyung Lee, Jinsu Kim, Jeongwoo Lee, Seongho Lee, Eui-Chan Jeon

**Affiliations:** 1 Department of Earth and Environmental Sciences, Sejong University, 98 Gunja-dong Gwangjin-gu, Seoul, 143-747 South Korea; 2 Cooperate Course for Climate Change, Sejong University, 98 Gunja-dong Gwangjin-gu, Seoul, 143-747 South Korea; 3 Department of Environment & Energy, Sejong University, 98 Gunja-dong Gwangjin-gu, Seoul, 143-747 South Korea

**Keywords:** Greenhouse gas, Methane, Nitrous oxide, Emission factor, Non-CO_2_, Power plant

## Abstract

In this research, in order to develop technology/country-specific emission factors of methane (CH_4_) and nitrous oxide (N_2_O), a total of 585 samples from eight gas-fired turbine combined cycle (GTCC) power plants were measured and analyzed. The research found that the emission factor for CH_4_ stood at “0.82 kg/TJ”, which was an 18 % lower than the emission factor for liquefied natural gas (LNG) GTCC “1 kg/TJ” presented by Intergovernmental Panel on Climate Change (IPCC). The result was 8 % up when compared with the emission factor of Japan which stands at “0.75 kg/TJ”. The emission factor for N_2_O was “0.65 kg/TJ”, which is significantly lower than “3 kg/TJ” of the emission factor for LNG GTCC presented by IPCC, but over six times higher than the default N_2_O emission factor of LNG. The evaluation of uncertainty was conducted based on the estimated non-CO_2_ emission factors, and the ranges of uncertainty for CH_4_ and N_2_O were between −12.96 and +13.89 %, and −11.43 and +12.86 %, respectively, which is significantly lower than uncertainties presented by IPCC. These differences proved that non-CO_2_ emissions can change depending on combustion technologies; therefore, it is vital to establish country/technology-specific emission factors.

## Introduction

In 2009, Korea announced greenhouse gas mitigation commitment to release 30 % less greenhouse gas than the “Business As Usual” level by 2020 (The Ministry of Environment 2010). It also enacted the “Low Carbon Green Growth Act” in 2010, setting legal grounds for regulation of greenhouse gas emissions to achieve the reduction target. And through the enforcement ordinance of the law, it implemented “GHG Target Management” in April 2010, a system that sets and manages greenhouse gas reduction and energy saving targets in large-scale work sites. In August 2010, 460 companies were designated by the government under these circumstances, so that they fall under government control. As of 2007, the companies released 380 million tons of CO_2_, which accounts for 60 % of the total greenhouse gas emissions in the country. In addition, it established the guidelines for the GHG Target Management in March 2011 (The Ministry of Environment 2011).

Although there is no globally regulated measurable, reportable and verifiable (MRV) system, Annex Ι countries set a rigorous MRV system at the national level (KIIEP 2009). Also, at the 15th Conference of the Parties, the Copenhagen Accord agreed that Non-Annex Ι countries should be required to establish their own MRV system and submit a country report every 2 years (UNFCCC 2009). Thus, Non-Annex Ι countries do not necessarily have to build the same kind of MRV system on the level of Annex Ι countries, but without an MRV system at the international level, they are highly likely not to be recognized in terms of greenhouse gas emission performance among other countries. In that regard, it is meaningful that Korea established a greenhouse gas MRV system.

At this juncture, Korea is in urgent need to secure well-documented data for drawing up a greenhouse gas inventory. Greenhouse gas emissions are characterized by different kinds of emissions, fuel, types of boilers, antipollution facilities, load factors, and other inherent factors. Non-CO_2_, in particular, is affected by combustion conditions, operational conditions, technological factors, and several other unknown factors (IPCC 2006; WRI/WBCSD 2005). Therefore, the Intergovernmental Panel on Climate Change (IPCC) recommends that each country put a priority on country- or technology-specific emission factors over default emission factors provided by the IPCC in calculating the amount of greenhouse gas emissions (IPCC 2006; Quick and Glick 2000). Despite this, Korea uses the default emission factors provided by that IPCC since it lacks its own research findings. As of 2006, greenhouse gas emissions by the energy sector has taken up 84 % of the total greenhouse gas emissions, 30 % (about 35 % of the total energy sector) of which have gone to the power generation sector. Thus, the country is expected to be enormously affected if greenhouse gas reduction obligation is imposed (Young-sung et al. 2006). In addition, according to long-term emission prospects, greenhouse gas emissions by the power generation sector are expected to reach 35 % of the total greenhouse gas emissions by 2020. In Korea’s case, a standard and system to classify power generation methods, fuel, combined cycle power generation, cogeneration, etc. should first be established, and greenhouse gas emission factors calculated based on that system.

In this regard, this paper analyzed liquefied natural gas (LNG) gas used in combined cycle power plants in Korea, calculating CH_4_ and N_2_O emission factors by measuring non-CO_2_ greenhouse gasses.

## Research method

We researched gas-fired combined cycle power plants in Korea using LNG as an energy source among energy industry, accounting for about 24 % of power generation capacity in Korea.

For CO_2_, emissions can be fairly estimated based on the amount of fuels combusted and the averaged carbon content of fuel because it mainly depend upon the carbon content of the fuel. However, emissions of non-CO_2_ are influenced by numerous additional factors such as combustion technology and operating conditions.

Therefore, in this study, to identify the emission characteristics of CH_4_ and N_2_O and develop emission factors from gas-fired turbine combined cycle power plants, eight plants were selected (Table 1).

**Table 1:** The combined cycle power plants investigated in this study (2007. 1. 1.–12. 31.)

Power plants (unit)	Generation capacity (kW)	Gross generation (MWh)	Average load (kW)	Peak load (kW)
Ilsan	900,000	3,568,156	407,324	817,372
Bundang	900,000	3,791,479	432,817	933,000
Anyang	450,000	1,646,561	187,964	506,000
Bucheon	450,000	1,562,279	178,342	500,000
Pyongtaek	480,000	921,916	105,242	524,000
Seoincheon	1,800,000	11,012,625	1,257,149	2,070,000
Sinincheon	1,800,000	13,004,005	1,484,475	2,028,000
GS Bugog	500,750	2,810,883	320,877	545,555
Total	7,280,750	38,317,904	4,374,190	7,923,927

Source: 2008 Statistics of Electric Power in Korea, KEPCO

### Sampling method

**Fig. 1:**
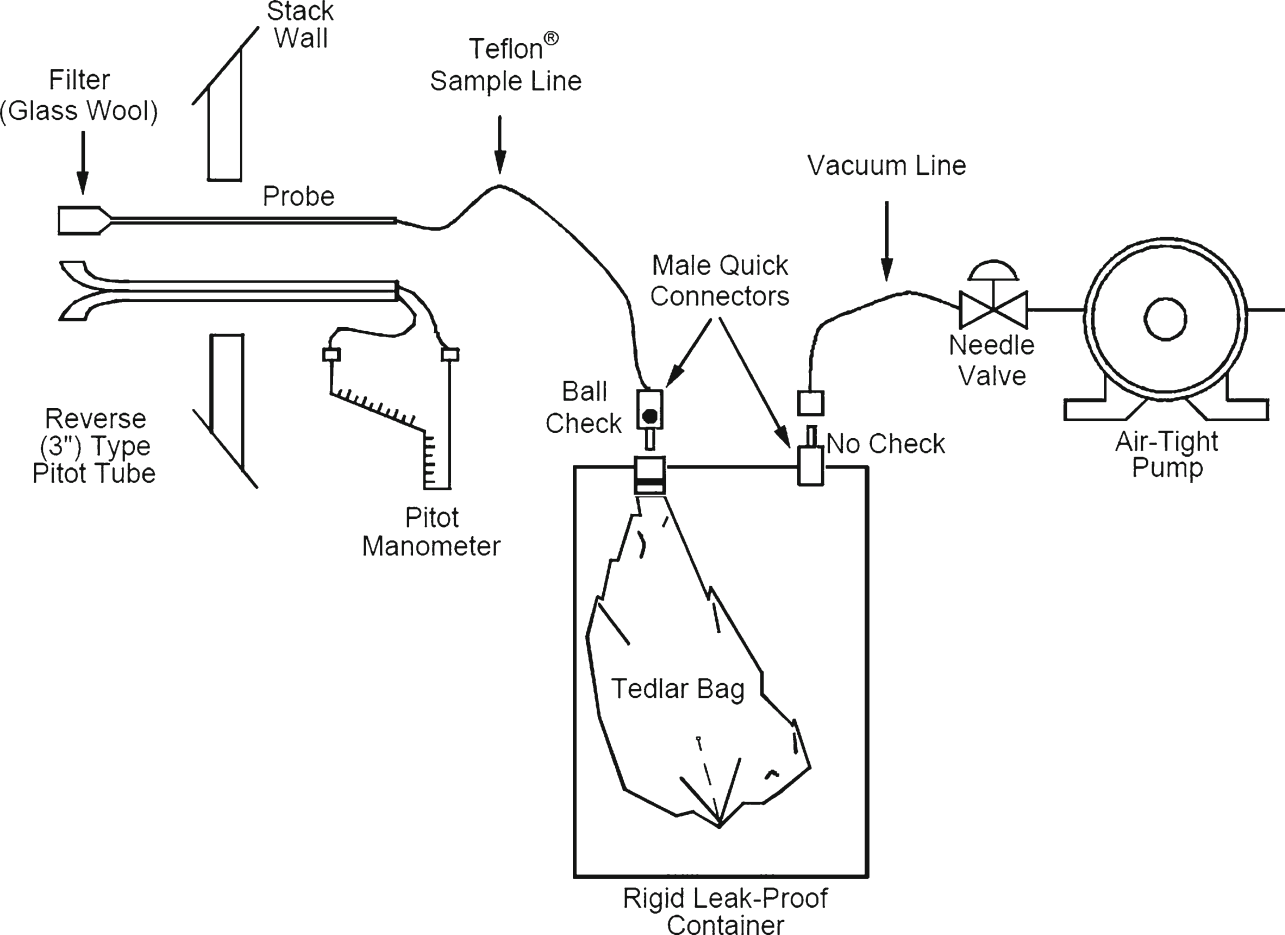
Diagram of greenhouse gas sampling system

When taking greenhouse gas samples, this paper used 1 L Tedlar bags (SKC, USA) as seen in Fig. 1 and applied United States Environmental Protection Agency (EPA 2001) Method 18. In order to reduce errors, we took three samples in succession on every occasion and measured exhaust gas temperature, moisture amount, flow velocity, pressure, and temperature as well (The Ministry of Environment 2004: Wight 1994). This paper took 585 samples from stacks of generators under operation at eight power plants across Korea.

### Exhaust gas analysis method

The concentrations of non-CO_2_ in exhaust gasses were analyzed by taking samples of exhaust gasses using a Tedlar bag, then analyzing them by ingredient in the laboratory. The quantitative concentration of non-CO_2_ was measured with gas chromatography (Model CP-3800, Varian, USA). Flame ionization detector (FID) and electrochemical detectors (ECD) were used as a detector; FID for analyzing CH_4_ and ECD for N_2_O. We used 1 and 3 m long Porapack QX 80/100 mesh column (stainless steel, external diameter of 3.175 mm, produced by Restek). The temperature of the injector, oven, and detector on the FID was set at 120, 70, and 250 °C, respectively. Additionally, the temperature of the injector, oven, and detector on the ECD was set at 120, 70, and 320 °C, respectively. Ultrapure nitrogen (99.9999 %) was used as carrier gas. When injecting the sample, we used 10-, 6-, and 4-port gas-switching valves to eliminate oxygen and moisture.

In order to carry out quantitative analysis of CH_4_ and N_2_O, we drew up calibration curves of each ingredient in advance and used them in calculating concentrations. The CH_4_ calibration curve was drawn up by measuring five samples with different concentrations within the range of 0.25–5.0 μmol/mol. The N_2_O calibration curve was drawn up by measuring five samples of different concentrations within the range of 0.5–10.0 μmol/mol. As a result, the *R* ^2^ value of CH_4_ and N_2_O was 0.9994 and 0.9992, respectively, showing high correlation.

### Moisture measurement method

Moisture extracting equipment (M-5, Astek Korea) and an electronic scale (Ohaus Adventurer, USA) was used to measure the amount of moisture in the exhaust gas. The temperature of the sample extracting equipment was maintained at 120 °C, while heat rays were quipped in the sample extracting pipe as the moisture in the exhaust gas condensed inside. In order to measure amount of moisture, we filled a cylindrical absorption bottle with anhydrous calcium chloride (Duksan, Korea) and connected it to a sample extracting pipe designed to collect greenhouse gasses. The amount of gas collected was measured to two decimal places (EPA method 4) with an integrating flow meter attached to the moisture extracting equipment. After collecting the sample, we closed the bottle with a stopper and measured the weight. Then, we calculated the moisture amount in the exhaust gas by applying the weight difference of the bottle before and after collecting the sample, flux collected, and gas temperature.

### Quality control of analyzing equipment (QA/AC)

To confirm the reproducibility of exhaust gas, for CH_4_ we analyzed the standard gas (RIGAS, Korea) of 1.1 μmol/mol concentration 10 times repetitively. As for N_2_O, we analyzed the standard gas (RIGAS) of 1.0 μmol/mol 10 times repetitively. The results of reproducibility analysis are presented in Table 2. CH_4_ and N_2_O showed excellent reproducibility with 0.19340 % and 0.57101 % relative standard error, respectively. The method detection limit of CH_4_ and N_2_O were 0.0558 and 0.0280 ppm, which are indicated on FID and ECD (see Table 3).

**Table 2:** Repeatability test of concentration analysis using CH_4_ and N_2_O standard gas

Times	Concentration (μmol/mol)	
	CH_4_	N_2_O
Standard^a^	1.1	1
1	1.11226	1.03801
2	1.10572	1.01238
3	1.10449	1.01570
4	1.10930	0.98851
5	1.09610	1.01171
6	1.09919	0.98571
7	1.09141	0.98551
8	1.09956	1.00819
9	1.09746	0.98588
10	1.09437	0.98644
Mean	1.10099	1.00180
SD	0.00673	0.01809
RSD (%)	0.61160	1.80570
SE	0.00213	0.00572
RSE (%)	0.19340	0.57101

*RSE* relative standard error

^a^Origin concentration of standard gas

**Table 3:** MDL values of GC/FID for CH_4_ and GC/ECD for N_2_O in this study

	CH_4_ (FID)		N_2_O (ECD)	
	Area	Concentration (ppm)	Area	Concentration (ppm)
1	536	0.0608	1,235	0.0329
2	612	0.0695	1,618	0.0430
3	952	0.1081	1,343	0.0357
4	861	0.0977	1,854	0.0493
5	508	0.0577	1,538	0.0409
6	742	0.0842	2,168	0.0577
7	672	0.0763	1,947	0.0518
SD		0.0187		0.0089
MDL		0.0558		0.0280

*MDL* method detection limit

### Calculation method of emission factor of non-CO_2_ emissions

**Fig. 2:**
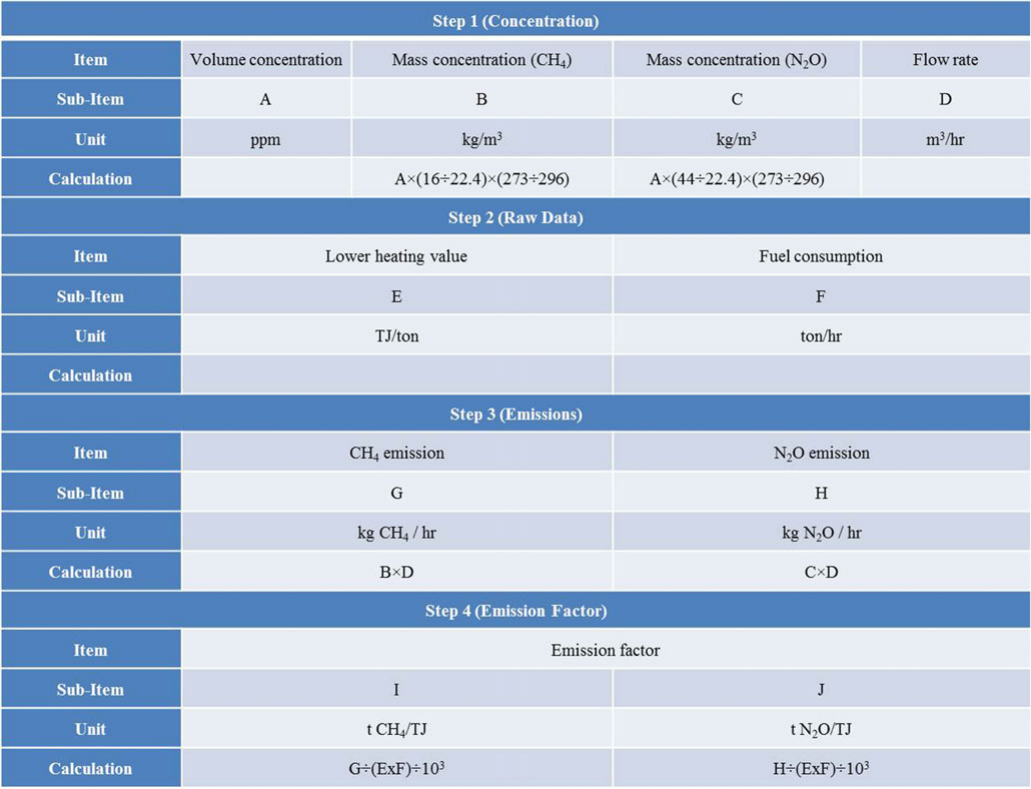
Calculation work-sheet to get non-CO2 emission factor by exhaust gas analysis in this study

In the case of CO_2_, element analysis of fuel produces a highly reliable emission factor. However, the emission characteristics of non-CO_2_ differ depending on combustion conditions, such as combustion technology. Therefore, it is difficult to use the emission factor produced from fuel analysis (IPCC 2006) as a representative value. Thus, this paper measured the emission gas concentration of power plants to calculate the non-CO_2_ emission factor. The work sheet (see Fig. 2) for calculating the emission factor through actual measurements consists of four steps. First is entering the non-CO_2_ concentration and flux, and conducting unit conversion to calculate the emission factor. Second is standardizing the energy unit of the fuel consumed. The third step is entering the amount of fuel consumed, as well as the electricity and heat produced. For the fourth step, the amount of non-CO_2_ emissions is calculated by entering the heating value of the fuel after analyzing it, and as a result, the non-CO_2_ emission factor is calculated.

## Result and discussion

### Non-CO_2_ emission characteristics

Table 4 shows the non-CO_2_ emission concentrations of each power plant. The samples were collected from the units of the power plants under operation and 15 samples were taken, on average, from each stack.

**Table 4:** Non-CO_2_ concentration from stacks in the combined cycle power plants

Plants	Capacity (MW)	Concentration (ppm)		Samples
		CH_4_	N_2_O	
A	≥100	2.33	0.55	Average of 90 samples
B	≥77	1.42	0.27	Average of 105 samples
C	≥75	1.53	0.36	Average of 30 samples
D	≥100	2.24	0.41	Average of 45 samples
E	≥80	1.56	0.43	Average of 45 samples
F	≥75	2.06	0.47	Average of 30 samples
G	≥150	2.12	0.49	Average of 120 samples
H	≥160	2.08	0.45	Average of 120 samples
Max		2.33	0.55	
Min		1.42	0.27	

The average CH_4_ concentration was 1.42–2.33 and 0.27–0.55 ppm for N_2_O. This is because each power plant has different operational conditions; the amount of fuel consumed per the amount of electricity generated and the emission flux of exhaust gas. For this paper, we researched the operational conditions, the amount of fuel consumed, and the emission flux at each time when samples were taken to calculate the non-CO_2_ emission factor of combined cycle power plants in Korea.

### Results of non-CO_2_ emission factor calculations

This paper first identified the characteristics of LNG fuel to calculate the non-CO_2_ emission factor of a combined cycle power plant that uses LNG as fuel. In order to apply non-CO_2_ emission concentrations and combustion conditions of exhaust gas, we used the amount of fuel consumed, and emission flux of TMS and the amount of electricity generated each time samples were taken to calculate the non-CO_2_ emission factor of each power plant. As shown in Table 5, which presents emission factors calculated in the research, the CH_4_ emission factor is 0.82 kg/TJ. This value was 18 % lower than the technology-specific CH_4_ emission factor of “combined cycle power plant using LNG as an energy source”, as IPCC suggests. In addition, the default emission factor of first fuel-based (tier 1 method of calculating emission amount) LNG suggested by the IPCC is 1 kg/TJ. The CH_4_ emission factor of this study is within the rage of the IPCC emission factor (0.3–3.0 kg/TJ). Japan researched 11 power plants to calculate the CH_4_ emission factor of its gas-fired turbine combined cycle (GTCC) power plants, and uses the average value of each power plant as a representative value. The CH_4_ emission factor of GTCC power plants in Japan was 0.75 kg/TJ (the emission factor of our research was 8 % higher). Finland’s case suggests different emission factors depending on the capacity of the GTCC power plant, using LNG as an energy source. The CH_4_ emission factor of a small sized GTCC power plant under the capacity of 5 MW is 3 kg/TJ, and that of a GTCC power plant over 5 MW is 1 kg/TJ, which indicates that non-CO_2_ emissions can be affected by the size of the facility.

**Table 5:** Non-CO_2_ emission factors of combined cycle power plant in this study

	Fuel type	Combustion technique/capacity	Emission factor (kg/TJ)		Remarks
			CH_4_	N_2_O	
This study	LNG	Combined cycle	0.82	0.65	Average of 8 facilities
2006 IPCC G/L^a^	LNG	Combined cycle	1	3	
2006 IPCC G/L^b^	LNG	–	1 (0.3–3)	0.1 (0.03–0.3)	
FINLAND NIR^c^	LNG	Gas turbine (including GTCC)/>5 MW	1	1	
		Gas turbine (including GTCC)/<5 MW	3	1	
Japan NIR^d^	Gaseous fuel	Gas turbine (including GTCC)	0.75	0.54	Average of 12 facilities

*GTCC* gas-fired turbine combined cycle

^a^2006 IPCC G/L—representative technology-specific default emission factor in utility source

^b^2006 IPCC G/L—default emission factors by only fuel type

^c^Greenhouse gas emissions in Finland 1990–2005 (Statistics Finland 2007)—mission factors of stationary sources

^d^National Greenhouse Gas Inventory Report of Japan (2007)—emission factors for different fuel and furnaces

The N_2_O emission factor of this study is 0.65 kg/TJ. This value is far lower than the technology-specific N_2_O emission factor of “combined cycle power plant using LNG as energy source”, as suggested by IPCC. However, the value is more than six times higher than the N_2_O basic emission factor of the first fuel-based (tier 1 method of calculating emission amount) LNG suggested by IPCC. The difference indicated that non-CO_2_ emissions change overwhelmingly by combustion technology, which is grounds for establishing country-specific or technology-specific emission factors. Japan researched 12 power plants to calculate the N_2_O emission factor of its GTCC power plants and uses 0.54 kg/TJ, the average value, as a country-specific emission factor. The value is 1/6 of the IPCC emission factor (combined cycle) and about 20 % lower than that of this study. In Finland’s case, the N_2_O emission factor of all GTCC power plants was 1 kg/TJ, which is 1/3 of the IPCC emission factor (combined cycle) and about 54 % higher than that of this study.

### Analyzing the measurement uncertainty of the non-CO_2_ emission factor

**Fig. 3:**
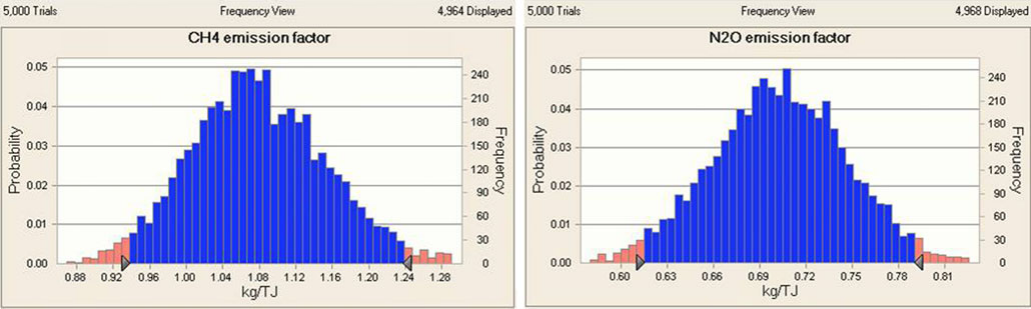
Results of simulation for non-CO2 emission factors in this study

This paper assessed uncertainty with the non-CO_2_ emission factor of GTCC power plants using LNG as an energy source with Monte-Carlo simulation. The assessment was conducted in accordance with the 2006 IPCC G/L and probability distributions of CH_4_ and N_2_O emission factors are selected as normal distribution because its *p* value was the highest among lognormal, gamma, uniform distribution. Figure 3 represents the simulation result of 5,000 repetitive analyses.

Table 6 shows the non-CO_2_ greenhouse gas emission factor and measurement uncertainty of GTCC power plants. The emission factor of CH_4_ is 0.82 kg/TJ and the lower and upper limits are set at 0.71 kg/TJ and 0.93 kg/TJ, respectively, with a 95 % confidence interval. In short, the uncertainty range of the CH_4_ emission factor is −12.96 to +13.89 %.

**Table 6:** Uncertainty range of non-CO_2_ emission factors estimated in this study (unit: percentage)

	CH_4_		N_2_O	
Combined cycle power plant	Distribution	Range	N_2_O	Range
IPCC	–	50–150	–	Oder of magnitude
Finland	Beta	−75–10	Beta	−75 to 10
This study	Normal	−12.96–13.89	Normal	−11.43 to 12.86

The average value of the N_2_O emission factor is 0.65 kg/TJ and the lower and upper limits are 0.58 and 0.73 kg/TJ, respectively, at a 95 % confidence interval. The uncertainty of the N_2_O emission factor is −11.43 % to +12.86 %.

## Conclusion

The results of the non-CO_2_ concentration in exhaust gas revealed that the average emission concentration of CH_4_ and N_2_O was 1.78 and 0.53 ppm, while the emission factors of CH_4_ and N_2_O calculated from non-CO_2_ concentration analysis were 0.82 and 0.65 kg/TJ, respectively.

The CH_4_ emission factor was 18 % lower than the technology-specific emission factor suggested by the IPCC. However, it was within the emission factor range (0.3–3 kg/TJ) of the fuel-based LNG. In comparison with other countries, the emission factor of this study was 8 % higher than that of Japan’s GTCC power plant and about 22 % lower than the emission factor of GTCC power plant in Finland with a capacity of more than 5 MW. On the other hand, the N_2_O emission factor was much lower than the technology-specific N_2_O emission factor suggested by the IPCC for combined cycle power plant using LNG as an energy source. But it was more than six times higher than the N_2_O emission factor of the fuel-based LNG suggested by the IPCC. In Japan’s case, the N_2_O emission factor of a GTCC power plant was 0.54 kg/TJ, and the emission factor of our study was 20 % higher than that. Meanwhile, the N_2_O emission factor of a GTCC power plant in Finland was 54 % higher than that of our research. And the ranges of uncertainty for CH_4_ and N_2_O were between −12.96 and +13.89 %, and −11.43 and +12.86 % respectively, which is significantly lower than uncertainties presented by IPCC. These differences of emission characteristics and precision of emission factors show us that non-CO_2_ emissions mainly depend on combustion technology, and there is a visible need for establishing country-specific or technology-specific emission factors. And these factors may lead to set up more reliable national greenhouse gas inventory following bottom up approach.

In order to calculate the exact amount of greenhouse gas emissions and set highly reliable greenhouse gas abatement goals, researches on various fuel and energy consuming facilities is needed to develop country-specific emission factors. Furthermore, for Korea to lead international negotiations (such as the Climate Change Convention), research should continue to set up accurate country-specific emission factors, which are used as an indicator in comparing and assessing the amount of greenhouse gas emissions and reduction.
